# The Function of the Odontoblasts

**Published:** 1891-03

**Authors:** J. E. Stanton

**Affiliations:** Boston, Mass.


### THE FUNCTION OF THE ODONTOBLASTS.[1]

BY J. E. STANTON, M.D., D.M.D., BOSTON, MASS.

About two months ago I had the pleasure of listening to a paper upon, and seeing some photographic views of, "New Formations in the Pulp-Cavity," or in the pulp itself. Dr. Cooke, of our Society, was the author of that paper, which, with the one read by Dr. Blaisdell and the discussion following, suggested to me the subject upon which this present paper is founded. In many of the specimens which Dr. Cooke presented, he observed and called attention to a membrane on the surface of the pulp. Dr. Cooke's specimens were all dried teeth which had been broken open for the purpose of finding the new deposits. This membrane was quite distinct from the rest of the pulp and the surrounding tooth, and you will easily recognize it as the membrana eboris, the ivory, or dentine-forming membrane, consisting of the layer of odontoblasts with which every pulp is surrounded. We all know that this layer consists of cells which are nucleated, each cell having three processes,—one to the deeper tissue of the pulp, another to the neighboring odontoblast, and the third running into the dentine, under the name of the dentinal fibril. We also know that it is through this odontoblastic layer that the tooth is formed, nourished, and kept in a healthy condition; in fact, this might be called the internal periosteum of the tooth. In the formation of the tooth the epithelium or epiblast dips down into the deeper layers in the form of a pear-shaped bulb, which becomes invaginated, and ultimately becomes the enamel-forming organ. Coming up from the mesoblastic stratum, which is the middle layer of the embryo, are cells in the form of a pyramid which fit into the invaginated portion of the epiblastic layer. This is the future tooth; strictly speaking, it is the first indication of dentine, and dentine is really the tooth, since it retains the shape so exactly after the enamel and cement are removed that we are enabled to name it correctly. This layer of cells proceeds at once to deposit the lime which it finds in the circulation and gradually builds up the form to its type limitation. As the tooth is developed from without inward, the odontoblasts recede until ultimately in advanced age the entire pulp is converted into dentine.

Is it not possible for us to treat exposed and diseased pulps

---

[1] Read before the Harvard Odontological Society, June 23, 1890.

more intelligently if we bear in mind, during the application of our remedies and coverings, this proper function of the odontoblastic layer, and regard it as surgeons do the periosteum of bone? They are careful to preserve that bone-forming and repairing membrane when they wish to treat a diseased bone, while we with many of the applications made to the surface of an exposed pulp will destroy the layer of odontoblasts which are the ivory-formers and repairers of the tooth. In a very large proportion of the cases shown by Dr. Cooke, the teeth were either filled, had cavities of decay, or had been injured by being clasped for the purpose of retaining artificial dentures. The injury was a deep one, resulting in all cases, so far as I was able to judge, in the formation of pulp-stones, nodules, or the conversion of the entire pulp into a mass of unorganized lime. In all cases the injury resulted in the formation of lime deposits in the substance of the pulp. From this one might infer that the amount deposited was in proportion to the length of the life of the pulp after receiving the shock which ultimately paralyzed the odontoblasts, and prevented them from converting the lime which was carried to them into normal dentine.

The pulp is peculiar. There is no other organ in the body like it; it has hardly any regular anatomical organization; there are no lymphatics to remove the products of disease or injury, and whatever change takes place in it is brought about by the agency of the odontoblasts, for, exclusive of these cells, the pulp substance is little more than embryonal tissue. This being the case, how careful we should be not to injure, by harsh fillings, pressure, or irritating applications, the only portion of the pulp which is capable of self-restoration. Is it not possible for us to attain greater success in the treatment of exposed pulps, if we consider them as we do other inflamed tissue, which needs soothing applications and free ventilation until the inflammation is subdued, rather than by sealing them up, or by flooding the odontoblasts with some escharotic that destroys these delicate cells? I will not attempt to outline a successful treatment, but trust some of us will get an idea from what I have said which may modify the present doubtful treatment of exposed pulps, or replace it by a more certain and successful one.

